# Sofosbuvir-Based Direct-Acting Antiviral Therapies for HCV in People Receiving Opioid Substitution Therapy: An Analysis of Phase 3 Studies

**DOI:** 10.1093/ofid/ofy001

**Published:** 2018-02-09

**Authors:** Jason Grebely, Jordan J Feld, David Wyles, Mark Sulkowski, Liyun Ni, Joe Llewellyn, Heshaam M Mir, Nika Sajed, Luisa M Stamm, Robert H Hyland, John McNally, Diana M Brainard, Ira Jacobson, Stefan Zeuzem, Marc Bourlière, Graham Foster, Nezam Afdhal, Gregory J Dore

**Affiliations:** 1 The Kirby Institute, UNSW Sydney, Sydney, New South Wales, Australia; 2 Toronto Centre for Liver Disease, Toronto, Ontario, Canada; 3 Division of Infectious Diseases, Denver Health and Hospital Authority, Denver, Colorado; 4 Johns Hopkins University, Baltimore, Maryl; 5 Gilead Sciences, Inc., Foster City, California; 6 Mount Sinai Beth Israel, New York, New York; 7 Johann Wolfgang Goethe University Medical Center, Frankfurt am Main, Germany; 8 Hospital Saint Joseph, Marseille, France; 9 Queen Mary University London, London, United Kingdom; 10 Beth Israel Deaconess Medical Center, Boston, Massachusetts

**Keywords:** DAA, drug use, hepatitis C virus, interferon-free, ledipasvir, PWID, sofosbuvir, velpatasvir, voxilaprevir

## Abstract

**Background:**

Hepatitis C virus (HCV) direct-acting antiviral therapy is effective among people receiving opioid substitution therapy (OST), but studies are limited by small numbers of nongenotype 1 (GT1) patients. The aim of this study was to evaluate the treatment completion, adherence, SVR12, and safety of sofosbuvir-based therapies in HCV patients receiving and not receiving OST.

**Methods:**

Ten phase 3 studies of sofosbuvir-based regimens included ION (ledipasvir/sofosbuvir ± ribavirin for 8, 12, or 24 weeks in GT1), ASTRAL (sofosbuvir/velpatasvir for 12 weeks in GT1-6), and POLARIS (sofosbuvir/velpatasvir and sofosbuvir/velpatasvir/voxilaprevir in GT1-6). Patients with clinically significant drug use (last 12 months) or noncannabinoids detected at screening were ineligible.

**Results:**

Among 4743 patients, 4% (n = 194) were receiving OST (methadone; n = 113; buprenorphine, n = 75; other, n = 6). Compared with those not receiving OST (n = 4549), those receiving OST (n = 194) were younger (mean age, 48 vs 54), more often male (73% vs 61%), GT3 (38% vs 17%), treatment-naïve (78% vs 65%), and cirrhotic (36% vs 23%). Among those receiving and not receiving OST, there was no significant difference in treatment completion (97% vs 99%, *P* = .06), SVR12 (94% vs 97%, *P* = .06), relapse (0.5% vs 2.1%, *P* = .19), adverse events (78% vs 77%, *P* = .79), or serious adverse events (3.6% vs 2.4%, *P* = .24). There was no difference in SVR12 in patients with cirrhosis (99% vs 95%, *P* = .25) or those with G3 (95% vs 95%, *P* = .77) in those receiving OST. Among patients receiving OST, SVR12 was high among those receiving methadone (95%) and buprenorphine (96%).

**Conclusion:**

Sofosbuvir-based therapies are effective and safe in patients receiving OST.

People who inject drugs (PWID) are disproportionately affected by hepatitis C virus (HCV) infection [1, 2]. Despite increasing liver-related morbidity and mortality among PWID [2, 3], some clinicians are reluctant to prescribe direct-acting antiviral (DAA) therapy for PWID or people receiving opioid substitution therapy (OST) based on concerns of poor adherence, lower response to therapy, and high rates of reinfection [4]. This is inconsistent with international guidelines recommending that all people should receive HCV treatment and that PWID should be prioritized, given the potential to reduce transmission to others [5–8]. Further data on treatment outcomes among populations of PWID receiving DAA therapy, including those receiving OST, are needed to change HCV health policy and clinical practice.

People with a history of injecting drug use include former injectors who have ceased injecting and recent PWID [9]. Some people with a history of injecting drug use may also be receiving OST (eg, methadone, buprenorphine) for the management of opioid dependence. Interferon-based therapy is effective in people with a history of injecting drug use, including those with recent injecting drug use and those receiving OST, with responses similar to that observed in large clinical studies [10–12]. Although data are emerging on outcomes to DAA-based HCV therapy among PWID receiving OST [13–23], most studies are limited by small numbers of HCV nongenotype 1 patients. There are also no published studies on the efficacy and safety of sofosbuvir/velpatasvir/voxilaprevir in people receiving OST.

Phase 3 studies of sofosbuvir-based therapy (the ION, ASTRAL, and POLARIS studies) included the evaluation of ledipasvir/sofosbuvir with or without ribavirin, sofosbuvir/velpatasvir, and sofosbuvir/velpatasvir/voxilaprevir in patients with chronic HCV genotype 1–6 [24–30]. People receiving stable OST were eligible for inclusion, but people with clinically relevant illicit drug use within 12 months of screening or illicit drugs (excluding cannabinoids) detected by a urine drug test at screening were excluded from study participation. These clinical trial populations are highly selected and may not be representative of recent PWID populations. However, studies in these populations contribute to the growing body of evidence on interferon-free DAA therapy among people receiving OST, particularly people with HCV nongenotype 1 infection.

The aim of this post hoc analysis of the phase 3 studies of sofosbuvir-based therapy was to evaluate the treatment completion, adherence, SVR12, and safety of sofosbuvir-based therapy in patients receiving OST and not receiving OST.

## METHODS

### Study Participants and Design

From October 2012 to May 2016, participants were enrolled in 10 multicenter, randomized clinical studies, including ION-1–3 (ClinicalTrials.gov identifier: NCT01701401, NCT01768286, and NCT01851330, respectively [24–26]), ASTRAL-1–3 (ClinicalTrials.gov: NCT02201940, NCT02220998, and NCT02201953, respectively [27, 28]), and POLARIS-1–4 (ClinicalTrials.gov: NCT02607735, NCT02607800, NCT02639338, and NCT02639247 [29, 30]).

In the ION-1–3 studies, a fixed-dose combination tablet of ledipasvir/sofosbuvir 90 mg/400 mg was administered for 8, 12, or 24 weeks with or without ribavirin in patients with chronic HCV genotype 1 infection [24–26]. Twice-daily ribavirin dose was given according to body weight (1000 mg daily < 75 kg or 1200 mg daily ≥ 75 kg). In the ASTRAL-1–3 studies, a fixed-dose combination tablet of sofosbuvir/velpatasvir 400 mg/100 mg was administered for 12 weeks in patients with chronic HCV genotypes 1–6 [27, 28]. In the POLARIS studies, a fixed-dose combination tablet of sofosbuvir/velpatasvir 400 mg/100 mg was administered for 12 weeks or a fixed-dose combination tablet of sofosbuvir/velpatasvir/voxilaprevir 400 mg/100 mg/100 mg was administered for 8 or 12 weeks in patients with chronic HCV genotypes 1–6 [29, 30].

Participants receiving OST (eg, methadone or buprenorphine with and without naloxone) were eligible for inclusion (OST determined based on reported concomitant medications). Patients were excluded from enrollment in these studies if they had clinically significant drug use within 12 months of screening (as assessed by the investigator based on participant self-report or medical chart review) or illicit drug use (excluding cannabinoids) detected by a urine drug test during the screening phase that was not explained by a prescription medication. The designs and results of these studies have been described previously [24–30].

### Study End Points

In this analysis, the end points included treatment completion, adherence (≥90% of doses), SVR12, safety (adverse events [AEs], serious AEs, and hemoglobin level <10 g/dL), and reinfection. The analyzed population included all randomized patients who received ≥1 dose of study medication. Adherence was measured by counting the number of unused tablets in the returned bottles to derive the number of administrated tablets. In situations where a bottle was not returned, the number of tablets administered from that bottle will be assumed to be 0. Adherence was calculated by dividing the number of total doses administered during therapy (determined by pill counts at week 4, 8, 12, 16, and 24 [where applicable] study visits) by the total expected number of prescribed doses. SVR12 was defined as the absence of quantifiable HCV RNA in serum (<25 IU/mL or <15 IU/mL), measured by COBAS TaqMan HCV Test, v2.0 (Roche Molecular Systems), at 12 weeks after the end of study treatment. Participants were monitored for recurrence (viral relapse or reinfection) at 4 weeks, 12 weeks (SVR12), and 24 weeks (SVR24) following the completion of treatment. Deep sequencing of the HCV NS5A and NS5B genes was performed for all patients at baseline and again for all patients with virologic failure in samples obtained at the first time point of failure with an HCV RNA >1000 IU/mL [24–30]. Phylogenetic analyses were used to distinguish viral relapse from reinfection.

### Statistical Analysis

Descriptive statistics, including means, frequencies, and percentages (with 95% confidence intervals [CIs] for SVR12) were used to summarize the data. The proportion of participants with treatment completion, ≥90% adherence, SVR12, and AEs was compared among people receiving and not receiving OST. Comparisons were made using a 2-sided Fisher exact test. All *P* values are 2-sided; a level of .05 was considered statistically significant. Statistical analysis was performed using SAS 9.4 software (SAS Institute Inc., Cary, NC).

## RESULTS

### Participant Characteristics

Overall, 4743 patients were enrolled and treated in the ION (n = 1952; ION-1, n = 865; ION-2, n = 440; ION-3, n = 647), ASTRAL (n = 1035; ASTRAL-1, n = 624; ASTRAL-2, n = 134; ASTRAL-3, n = 277), and POLARIS studies (n = 1756; POLARIS-1, n = 263; POLARIS-2, n = 941; POLARIS-3, n = 333; POLARIS-4, n = 219). Among individuals in all these studies, 4% (n = 194) were receiving OST at enrollment.

The clinical characteristics of the study participants are shown in Table 1. Among patients receiving OST (n = 194), 27% (n = 53) received ledipasvir/sofosbuvir with or without ribavirin (for 8, 12, or 24 weeks), 47% (n = 92) received sofosbuvir/velpatasvir for 12 weeks, and 25% (n = 49) received sofosbuvir/velpatasvir/voxilaprevir (for 8 or 12 weeks). Among patients not receiving OST (n = 4549), 42% (n = 1899) received ledipasvir/sofosbuvir with or without ribavirin (for 8, 12, or 24 weeks), 36% (n = 1643) received sofosbuvir/velpatasvir for 12 weeks, and 22% (n = 1007) received sofosbuvir/velpatasvir/voxilaprevir (for 8 or 12 weeks).

**Table 1.** **Baseline Demographic and Clinical Characteristics of Patients With Chronic HCV Infection Receiving Sofosbuvir-Based Therapies in the ION, ASTRAL, and POLARIS Phase 3 Clinical Trials, by Receipt of Opioid Substitution Therapy**

Characteristic	OST at Enrollment (n = 194), n (%)	No OST at Enrollment (n = 4549), n (%)
Mean (SD) age, y	48 (10.7)	54 (10.4)
Male sex, n (%)	141 (73)	2770 (61)
HCV genotype, n (%)^a^		
1a	84 (43)	2109 (46)
1b	12 (6)	816 (18)
2	14 (7)	409 (9)
3	74 (38)	787 (17)
4	10 (5)	269 (6)
5	0	54 (1)
6	0	86 (2)
HCV RNA log_10_ IU/mL, mean (SD)	6.3 (0.7)	6.3 (0.7)
HCV RNA ≥ 800000 IU/mL, n (%)	142 (73)	3456 (76)
Fibrosis stage		
F0	42 (22)	826 (18)
F1	23 (12)	410 (9)
F2	45 (24)	1141 (25)
F3	30 (16)	721 (16)
F4	51 (27)	1410 (31)
Treatment-experienced, n (%)	42 (22)	1568 (34)
Therapy		
Ledipasvir/sofosbuvir ± ribavirin (8 wk)	8 (4)	423 (9)
Ledipasvir/sofosbuvir ± ribavirin (12 wk)	32 (16)	835 (18)
Ledipasvir/sofosbuvir ± ribavirin (24 wk)	13 (7)	641 (14)
Sofosbuvir/velpatasvir (12 wk)	92 (47)	1643 (36)
Sofosbuvir/velpatasvir/voxilaprevir (8 wk)	41 (21)	570 (13)
Sofosbuvir/velpatasvir/voxilaprevir (12 wk)	8 (4)	437 (10)
OST, n (%)		
Methadone	113 (58)	-
Buprenorphine	35 (18)	-
Buprenorphine/naloxone	40 (21)	-
Other	6 (3)	-

Abbreviations: HCV, hepatitis C virus; OST, opioid substitution therapy.

^a^Nineteen patients were classified as other, unknown, or missing, and all were not receiving OST at enrollment.

Among patients receiving OST (n = 194), 36% (n = 70) had cirrhosis, 22% (n = 42) were treatment-experienced, and 38% (n = 74) were infected with HCV genotype 3. Among patients not receiving OST (n = 4549), 23% (n = 1041) had cirrhosis, 34% (n = 1568) were treatment-experienced, and 17% (n = 787) were infected with HCV genotype 3.

### HCV Treatment Completion and Adherence

The proportion of participants completing HCV therapy was 97.4% (189/194; 95% CI, 94.1%–99.2%) among participants receiving OST, compared with 98.9% (4501/4549; 95% CI, 98.6%–99.2%) among those not receiving OST (*P* = .064) (Table 2). The reasons for treatment discontinuation among patients receiving OST (n = 5) included AEs (n = 1), loss to follow-up (n = 1), consent withdrawal (n = 1), lack of efficacy (n = 1), and noncompliance (n = 1). The reasons for treatment discontinuation among patients not receiving OST (n = 48) included AEs (n = 19), loss to follow-up (n = 10), consent withdrawal (n = 6), protocol violation (n = 6), lack of efficacy (n = 4), noncompliance (n = 1), and pregnancy (n = 2). Among patients receiving OST, the proportion of participants completing therapy with ledipasvir/sofosbuvir with or without ribavirin was 96.2% (51/53), sofosbuvir/velpatasvir was 96.7% (89/92), and sofosbuvir/velpatasvir/voxilaprevir was 100% (49/49).

**Table 2.** Treatment and Safety Outcomes Among Patients With Chronic HCV Infection Receiving Sofosbuvir-Based Therapies in the ION, ASTRAL, and POLARIS Phase 3 Clinical Trials, by Receipt of Opioid Substitution Therapy

Characteristic	OST at Enrollment	No OST at Enrollment	*P*
Overall, n/N (%)			
Treatment completion	189/194 (97.4)	4501/4549 (98.9)	.064
≥90% adherence	175/194 (90.2)	4291/4549 (94.3)	.027
SVR12	183/194 (94.3)	4405/4549 (96.8)	.062
Adverse events	152/194 (78.4)	3517/4549 (77.3)	.79
Severe adverse events	7/194 (3.6)	108/4549 (2.4)	.24
Ledipasvir/sofosbuvir ± ribavirin			
Treatment completion	51/53 (96.2)	1863/1899 (98.1)	.28
≥90% adherence	47/53 (88.7)	1791/1899 (94.3)	.12
SVR12	49/53 (92.5)	1839/1899 (96.8)	.093
Adverse events	47/53 (88.7)	1513/1899 (79.7)	.12
Severe adverse events	2/53 (3.8)	50/1899 (2.6)	.65
Sofosbuvir/velpatasvir			
Treatment completion	89/92 (96.7)	1634/1643 (99.5)	.022
≥90% adherence	82/92 (89.1)	1559/1643 (94.9)	.029
SVR12	87/92 (94.6)	1601/1643 (97.4)	.099
Adverse events	68/92 (73.9)	1251/1643 (76.1)	.62
Severe adverse events	4/92 (4.3)	33/1643 (2.0)	.13
Sofosbuvir/velpatasvir/voxilaprevir			
Treatment completion, n (%)	49/49 (100.0)	1004/1007 (99.7)	1.00
≥90% adherence	46/49 (93.9)	941/1007 (93.4)	1.00
SVR12	47/49 (95.9)	965/1007 (95.8)	1.00
Adverse events	37/49 (75.5)	753/1007 (74.8)	1.00
Severe adverse events	1/49 (2.0)	25/1007 (2.5)	1.00

Abbreviations: HCV, hepatitis C virus; OST, opioid substitution therapy.

The proportion of participants with ≥90% adherence to therapy was 90.2% (175/194; 95% CI, 85.1%–94.0%) among participants receiving OST, compared with 94.3% (4291/4549; 95% CI, 93.6%–95.0%) among those not receiving OST (*P* = .027) (Table 2). Of the 19 patients receiving OST who had ˂90% calculated adherence, 12 patients achieved SVR12 and 7 patients failed to achieve SVR12 (3 were lost to follow-up, 1 withdrew consent on day 29, 1 had virologic breakthrough and drug levels consistent with nonadherence, 1 discontinued on day 1 due to AE, 1 was discontinued by the investigator on day 5 due to nonadherence). Among patients receiving OST, the proportion of participants with ≥90% adherence with ledipasvir/sofosbuvir with or without ribavirin was 88.7% (47/53), sofosbuvir/velpatasvir was 89.1% (82/92), and sofosbuvir/velpatasvir/voxilaprevir was 93.9% (46/49).

### HCV Treatment Outcomes

The proportion with SVR12 among those receiving OST was 94.3% (183/194; 95% CI, 90.1%–97.1%) compared with 96.8% for those not receiving OST (4405/4549; 95% CI, 96.3%–97.3%; *P* = .062) (Table 2). SVR12 by treatment type and duration for participants receiving and not receiving OST is shown in Table 2.

Among patients receiving OST, the proportion of participants with SVR12 with ledipasvir/sofosbuvir with or without ribavirin was 92.5% (49/53), sofosbuvir/velpatasvir was 94.6% (87/92), and sofosbuvir/velpatasvir/voxilaprevir was 95.9% (47/49). Further, among patients with HCV genotype 3, the response to therapy among patients receiving sofosbuvir/velpatasvir was 95.8% (46/48) and receiving sofosbuvir/velpatasvir/voxilaprevir was 92.3% (24/26).

Among patients receiving OST, the proportion of participants with SVR12 among people with F0 was 88.1% (37/42), with F1 was 91.3% (21/23), with F2 was 97.8% (44/45), with F3 was 93.3% (28/30), and with F4 was 98.0% (50/51).

Among patients receiving OST across treatment regimens, there was no difference in SVR12 in those receiving methadone and buprenorphine (94.7% vs 96.0%, *P* = 1.0), patients with and without cirrhosis (98.6% vs 91.9%, *P* = .089), and patients with genotype 3 as compared with genotype 1a (94.6% vs 95.2%, *P* = .850).

### Safety

The proportions with AEs (78.4%; 95% CI, 71.9%–83.9%; vs 77.3%; 95% CI, 76.1%–78.5%; *P* = .790) (Tables 2 and 3) and serious AEs (3.6%; 95% CI, 1.5%–7.3%; vs 2.4%; 95% CI, 2.0%–2.9%; *P* = .200) (Table 2) were similar among participants receiving and not receiving OST. AEs were mostly mild or moderate in severity.

**Table 3.** Adverse Events Among Patients With Chronic HCV Infection Receiving Sofosbuvir-Based Therapies in the ION, ASTRAL, and POLARIS Phase 3 Clinical Studies, by Receipt of Opioid Substitution Therapy

	OST at Enrollment			No OST at Enrollment		
Adverse Event, n (%)	Ledipasvir/ Sofosbuvir ± Ribavirin (n = 53)	Sofosbuvir/Velpatasvir (n = 92)	Sofosbuvir/Velpatasvir/ Voxilaprevir (n = 49)	Ledipasvir/ Sofosbuvir ± Ribavirin (n = 1899)	Sofosbuvir/Velpatasvir (n = 1643)	Sofosbuvir/Velpatasvir/ Voxilaprevir (n = 1007)
Adverse events in >10%						
Headache	12 (22.6)	20 (21.7)	8 (16.3)	443 (23.3)	450 (27.4)	269 (26.7)
Fatigue	19 (35.8)	18 (19.6)	11 (22.4)	556 (29.3)	364 (22.2)	222 (22.0)
Nausea	12 (22.6)	14 (15.2)	12 (24.5)	253 (13.3)	184 (11.2)	150 (14.9)
Diarrhea	4 (7.5)	7 (7.6)	5 (10.2)	151 (8.0)	110 (6.7)	183 (18.2)
Insomnia	5 (9.4)	5 (5.4)	3 (6.1)	232 (12.2)	112 (6.8)	59 (5.9)
Vomiting	4 (7.5)	6 (6.5)	6 (12.2)	60 (3.2)	42 (2.6)	24 (2.4)

Abbreviations: HCV, hepatitis C virus; OST, opioid substitution therapy.

### HCV Reinfection

Two patients were found to have reinfection with a different genotype than at baseline. Neither subject was receiving OST at baseline. One patient enrolled in ASTRAL-3 had genotype 3a at baseline and received SOF/VEL for 12 weeks. The patient achieved SVR4 and was found to have genotype 1a 12 weeks after the completion of therapy. Another patient enrolled in POLARIS-2 had genotype 1a and received SOF/VEL for 12 weeks. The patient achieved SVR12 but was found to have genotype 3a 24 weeks after therapy.

## DISCUSSION

This post hoc analysis of sofosbuvir-based therapies from the ION, ASTRAL, and POLARIS studies demonstrated high SVR12 rates among patients receiving OST, including those with HCV genotype 3 receiving sofosbuvir/velpatasvir and sofosbuvir/velpatasvir/voxilaprevir. Similar treatment completion, SVR12, and AE rates were observed among patients with chronic HCV genotypes 1–6 receiving and not receiving OST, although patients not receiving OST had a significantly higher proportion with ≥90% adherence. Collectively, these data add to the body of evidence supporting the efficacy and safety of DAA treatment for HCV among people receiving stable OST, consistent with international recommendations [5–8].

Overall, the SVR was 94% among patients receiving OST and sofosbuvir-based therapy, with no observed difference in response compared with those not receiving OST, which is consistent with previous post hoc analyses of the ION and ASTRAL studies [21, 22] and other studies in this population [23]. These results are also comparable with a large phase 3 study of people receiving stable OST (recent injecting drug use at screening was permitted) and HCV genotype 1, 4, and 6 infection receiving elbasvir/grazoprevir for 12 weeks, where an SVR of 91% was observed [20]. This study adds to the literature by providing further data among patients with cirrhosis and genotype 3 infection in people receiving OST, enabling a more precise estimate of outcomes in this population. Also, this is the first study to report outcomes with the combination of sofosbuvir/velpatasvir/voxilaprevir in patients stable on OST. These data are consistent with previous data demonstrating that interferon-based HCV therapy is safe and effective among people receiving OST [10–12, 31–33]. Collectively, these data support DAA therapy for patients stable on OST.

Treatment completion was high among people receiving OST (97%), with no difference between those not receiving OST, consistent with other studies of interferon-free [21, 22] and interferon-based therapy [10–12, 31, 32]. Adherence to therapy was significantly lower in people receiving OST as compared with those not receiving OST (90% vs 94%), although it is uncertain whether this would be clinically significant. In a meta-analysis of interferon-based studies among PWID, engagement in addiction treatment was associated with higher treatment completion [12]. Previous studies have demonstrated that the colocation of HCV services and drug treatment can be successfully integrated [34], with the colocation of HCV care in OST clinics welcomed by the large majority of participants and providers [35]. Further efforts are needed to expand the integration of HCV DAA therapy in drug and alcohol clinics and community health clinics that also provide OST. Also, improved education and training of practitioners working in drug treatment clinics about HCV testing, liver disease assessment, and HCV treatment are required to further develop competency and expand HCV treatment access for people receiving OST.

It is notable that there were no cases of HCV reinfection following DAA therapy among people receiving OST in the ION, ASTRAL, and POLARIS studies. Previous studies have demonstrated reinfection rates ranging from 1% to 5% per 100 person-years following successful interferon-based [10, 36–38] and DAA therapy [20] among people with a history of injecting drug use or those receiving OST. However, the sample size and duration of follow-up in this study are limited, and the included population is likely at lower risk of reinfection, given that they were not using illicit drugs at the time of treatment initiation. Further long-term studies of HCV reinfection among people receiving OST and recent PWID are required to more fully characterize the risk of HCV reinfection and associated risk factors.

This study has a number of limitations. People with active drug use at baseline were excluded from participating in the ION, ASTRAL, and POLARIS studies, and as such, enrolled participants represented a selected population likely to be engaged in care. Therefore, these findings may not be generalizable to other PWID populations (particularly those not receiving stable OST or recent PWID). Further, this was also a post hoc analysis, which was not defined prior to the initiation of these studies. Also, the data with respect to adherence must be interpreted with caution. Adherence in these studies was measured by counting the number of pills in returned pill bottles. In instances where participants did not return their pill bottles, a conservative measure of adherence was used and adherence for that period was assumed to be 0%. Given limited data on interferon-free treatment outcomes among people receiving OST (particularly people with cirrhosis and HCV genotype 3), these data still provide important guidance for HCV management in these populations.

In conclusion, these data demonstrate that sofosbuvir-based therapy is effective and well tolerated among patients receiving OST. Although this study provides important data to add to the literature on HCV therapy in people receiving OST, further data are still needed on DAA therapy among people with recent or ongoing injecting drug use. Ongoing clinical trials evaluating interferon-free therapy among PWID with recent drug use (SIMPLIFY, NCT02336139; HERO, NCT02824640) and PWID with recent drug use and/or those receiving OST (D3FEAT, NCT02498015) will hopefully provide further data in this regard. Global HCV elimination efforts will require the inclusion of PWID as a key priority population, and strategies are needed to enhance HCV care in this important group.
